# Dehydroepiandrosterone (DHEA) reduces embryo aneuploidy: direct evidence from preimplantation genetic screening (PGS)

**DOI:** 10.1186/1477-7827-8-140

**Published:** 2010-11-10

**Authors:** Norbert Gleicher, Andrea Weghofer, David H Barad

**Affiliations:** 1 Center for Human Reproduction (CHR) - New York and the Foundation for Reproductive Medicine, New York, NY, USA; 2 Department of Obstetrics, Gynecology and Reproductive Sciences, Yale University School of Medicine, New Haven, CT, USA; 3 Department of Obstetrics and Gynecology, Vienna University School of Medicine, Vienna, Austria; 4 Departments of Epidemiology and Social Medicine and Obstetrics, Gynecology and Women's Health, Albert Einstein College of Medicine, Bronx, NY, USA

## Abstract

**Background:**

Dehydroepiandrosterone (DHEA) has been reported to improve pregnancy chances in women with diminished ovarian reserve (DOR), and to reduce miscarriage rates by 50-80%. Such an effect is mathematically inconceivable without beneficial effects on embryo ploidy. This study, therefore, assesses effects of DHEA on embryo aneuploidy.

**Methods:**

In a 1:2, matched case control study 22 consecutive women with DOR, supplemented with DHEA, underwent preimplantation genetic screening (PGS) of embryos during in vitro fertilization (IVF) cycles. Each was matched by patient age and time period of IVF with two control IVF cycles without DHEA supplementation (n = 44). PGS was performed for chromosomes X, Y, 13, 16, 18, 21 and 22, and involved determination of numbers and percentages of aneuploid embryos.

**Results:**

DHEA supplementation to a significant degree reduced number (P = 0.029) and percentages (P < 0.001) of aneuploid embryos, adjusted for relevant covariates. Short term supplementation (4-12 weeks) resulted in greatest reduction in aneuploidy (21.6%, 95% CI -2.871-46.031).

**Discussion:**

Beneficial DHEA effects on DOR patients, at least partially, are the likely consequence of lower embryo aneuploidy. DHEA supplementation also deserves investigation in older fertile women, attempting to conceive, where a similar effect, potentially, could positively affect public health.

## Background

Dehydroepiandrosterone (DHEA) has been demonstrated to improve embryo quality and pregnancy chances in women with diminished ovarian reserve (DOR) [1-3]. How these effects are achieved is, however, unknown. A small pilot study of limited power suggested that DHEA may reduce aneuploidy [4]. Since aneuploidy in human embryos is frequent and increases with advancing female age [5,6], a reduction in aneuploidy could, at least partially, explain improved embryo quality and pregnancy rates.

Aneuploidy in preimplantation embryos can be demonstrated through preimplantation genetic screening (PGS) [7]. PGS is, however, only rarely indicated in women with DOR, where often only small embryo numbers are available, and embryo selection, therefore, does not offer clinical benefits [8]. PGS in such cases may, actually, reduce pregnancy chances with in vitro fertilization (IVF) [9].

Our initial pilot study, attempting to investigate DHEA effects on ploidy, was underpowered. In that study we were able to demonstrate that DHEA supplemented patients had greater chances of at least one euploid embryo. The study, likely because of small patient numbers, however, failed to demonstrate significant decreases in overall aneuploidy [4].

For lack of adequate PGS case numbers, we, therefore, pursued an alternative strategy by investigating miscarriage rates after DHEA supplementation as a surrogate for aneuploidy risk [10]. Since at least 60 percent of spontaneous pregnancy loss is attributable to chromosomal abnormalities [11], we hypothesized that significant reductions in aneuploidy after DHEA supplementation should be reflected in lower miscarriage rates. This was, indeed, confirmed in a study, involving patients from two independent centers in New York City and Toronto, Canada [10].

While results of this study were strongly supportive of a DHEA effect on aneuploidy, they were unable to offer direct evidence, which can only come from PGS studies of human embryos. Such a study is presented here.

## Methods

### Patient populations

We retrieved from our center's computerized research data bank a total of 22 consecutive DOR patients who underwent IVF/PGS while on DHEA supplementation. Only first IVF cycles were analyzed. These cycles were matched with the two control cycles not on DHEA supplementation, based on patient age and year of treatment (44 controls). Primary medical records for all of these patients were pulled and manually reviewed by one of the authors (A.W.).

A diagnosis of DOR was reached if patients demonstrated abnormally elevated age-specific baseline follicle stimulating hormone (FSH) or abnormally low age-specific anti-Müllerian hormone (AMH) levels. Normal age-specific hormone levels were defined by 95% confidence intervals at all ages, as previously reported [12,13]. Since patients with DOR were, thus, uniformly diagnosed before IVF cycle protocols were determined, they were all supplemented with DHEA, and stimulation adjustments were made. As we previously reported, this results in improved oocyte and embryo yields in comparison to patients who are not diagnosed with use of age-specific FSH and AMH [12,13].

### DHEA supplementation

During the study period all DOR patients at our center routinely received DHEA supplementation [14]. Those not receiving DHEA, therefore, by definition, had age-appropriate ovarian reserve, confirmed by normal anti-Müllerian hormone (AMH), follicle stimulating hormone (FSH) baseline levels and estradiol. Patients receiving DHEA supplementation were prescribed 25 mg of micronized, pharmaceutical grade DHEA, T.I.D, for at least four weeks prior to IVF cycle start [14]. Short-term supplementation was defined as 4 to 12 weeks of DHEA prior to IVF and PGS; supplementation beyond that was considered long-term supplementation.

### Ovarian stimulation

Patients with premature DOR, or if over 40 years of age, in first treatment cycles universally receive a so-called microdose gonadotropin releasing hormone agonist (GnRH-a) ovarian stimulation protocol, characterized by leuprolide acetat (50 μg/0.1 mL, b.i.d.; Lupron^®^, Abbot Pharmaceuticals, North Chicago, IL) and ovarian stimulation with follicle stimulating hormone (FSH, 300 IU-450 IU daily) and human menopausal gonadotropins (hMG, 150 IU). If under age 40 with normal ovarian reserve patients receive down regulation with full dose GnRH-a (1.0 mg/0.1 mL) and ovarian stimulation with up to 300 IU of gonadotropins, usually half as FSH and half as hMG. Higher luteinizing hormone (LH) contributions to ovarian stimulation, if anything, reduce embryo aneuploidy [15]. The small difference in ovarian stimulation protocols between women with DOR and controls, therefore, potentially biases study outcome towards lower aneuploidy rates in the control population, which received a proportionally higher LH contributions to ovarian stimulation.

### Preimplantation genetic screening (PGS)

PGS was performed, utilizing fluorescence in situ hybridization (FISH) in routine fashion, utilizing probes for seven chromosomes (X, Y, 13, 16, 18, 21 and 22) on day three after fertilization, when embryos reached six to eight cell stages. This restricted chromosome panel is currently routinely utilized for PGS [15].

### Statistical analysis

A general linear model was constructed to assess DHEA effects on percent aneuploidy after adjustment for age, indications for PGS, stimulation protocol and total gonadotropin dosage utilized. The latter adjustment was made as a surrogate for potential physician biases in how individual patients were stimulated, and potential effects such stimulation biases may have on ploidy [15,16].

All patients at our center sign a universal informed consent at time of initial presentation, which permits the extraction of clinical data from patient records as long as confidentiality of the record and anonymity of patients is maintained. The center's Institutional Review Board, therefore, permits such studies under expedited review.

## Results

### Patients

Table 1 summarizes characteristics of study and control patients. The two groups did not differ in age and race/ethnicity. DHEA patients were significantly more obese but expressed poorer ovarian reserve, based on lower AMH (P = 0.045) and significantly higher gonadotropin utilization (P = 0.002). Such a conclusion was also supported by trends towards higher FSH and smaller oocyte yields (9.6 ± 6.2 vs. 11.7 ± 6.3). Embryo numbers transferred (1.4 ± 0.9 vs. 1.5 ± 0.7), embryos cryopreserved (0.7 ± 1.6 vs. 0.6 ± 1.2), embryos undergoing PGS (7.3 ± 3.9 vs. 6.6 ± 3.6) and embryo grades (3.4 ± 0.4 vs. 3.5 ± 0.3) were similar between both groups.

**Table 1:** Patient characteristics

	DHEA	Controls	P-value
Number	22	44	
Age (years, Mean ± SD)	37.9 ± 4.7	37.2 ± 4.4	N.S.
Race/ethnicity (%)			
Caucasian	14 (63.6)	21 (47.7)	
African	2 (9.1)	3 (6.8)	
Asian	4 (18.2)	14 (31.8)	
Middle Eastern	1 (4.5)	6 (13.6)	
BMI (Mean ± SD)	24.4 ± 3.8	21.0 ± 1.7	0.006
AMH (ng/mL, mean ± SD)	1.3 ± 1.2	2.0 ± 1.9	0.045
Range	0.8 - 2.1	1.0 - 4.4	
FSH (mIU/mL, mean ± SD)	10.1 ± 6.8	8.0 ± 5.5	N.S.
Range	8.4 - 12.2	7.1 - 9.1	
Oocytes retrieved (n, mean ± SD)	9.6 ± 6.2	11.7 ± 6.3	N.S.
Embryos (n, mean ± SD)			
Transferred	1.4 ± 0.9	1.5 ± 0.7	
Cryopreserved	0.7 ± 1.6	0.6 ± 1.2	
Undergoing PGS	7.3 ± 3.9	6.6 ± 3.6	
Grades	3.4 ± 0.4	3.5 ± 0.3	
Total gonadotropins dosage (IU, mean ± SD)	5711 ± 1818	4048 ± 1886	0.002

^1 ^Difference in BMI was based on significant difference in weight (P = 0.02) not height (P = 0.24)

### Aneuploidy

**Figure 1:**
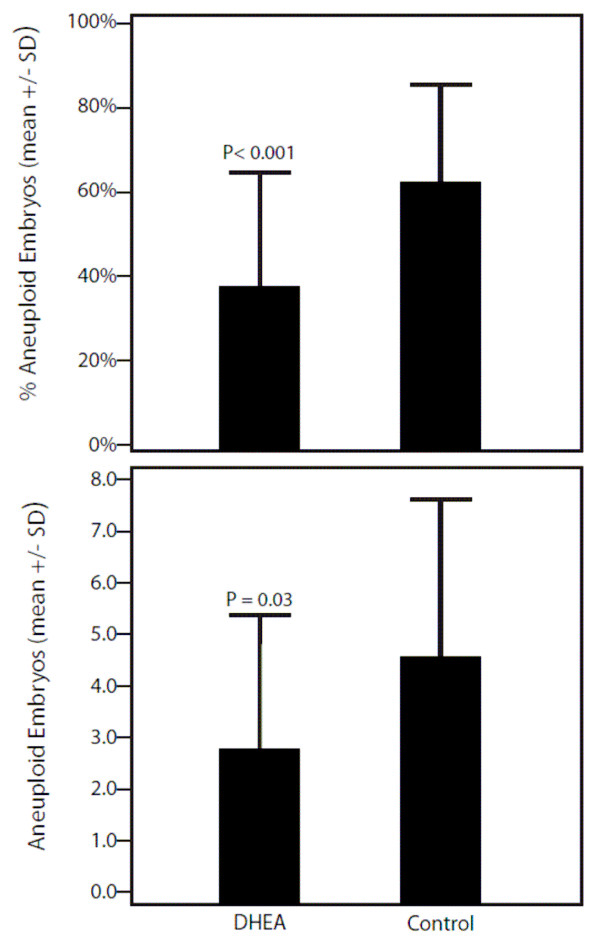
**Comparison of absolute and percentages of aneuploidy in DHEA and control patients**.

As Figure 1, however, demonstrates, aneuploid embryos were significantly more prevalent amongst controls (4.5 ± 3.1 vs. 2.8 ± 2.5; P = 0.03), as were percentages of aneuploidy (61.0 ± 22.4 vs. 38.2 ± 24.4; P < 0.001). In the general linear model, after adjustment for age, FSH dose and indication for PGS, the association of DHEA supplementation effects on ploidy remained significant (F = 13.2, df 1, p = 0.001). As expected, women who underwent PGS for aneuploidy screening had a greater percentage of aneuploidy embryos than women who underwent PGS for elective gender selection purposes (P < 0.007).

Possibly because of still relatively small study numbers, no specific aneuploidy pattern, affecting distinct chromosomes, was apparent.

Mean length of DHEA supplementation was 7.3 ± 2.2 weeks in the short and 19.1 ± 9.1 weeks in the long treatment group. Women in the short treatment group demonstrated the greatest reduction in aneuploidy (21.6%, 95%CI -2.871-46.031).

## Discussion

This study supports prior preliminary evidence that DHEA supplementation reduces aneuploidy in women with DOR, first suggested in a small pilot study, when at least one euploid embryo was found significantly more frequently after DHEA than in matched control cycles [4]. Subsequently, we also demonstrated that DHEA supplementation reduces miscarriage rates to a degree that cannot be explained without significant contribution from reduced aneuploidy [10].

By demonstrating no difference in embryo grades between DHEA and control cycles (Table 1), this study also demonstrates, once more, that embryo morphology, as currently routinely assessed in most IVF laboratories, does not reflect on embryo ploidy and, therefore, is limited in clinical value.

Since a majority of miscarriages are believed to be consequence of aneuploidy [11], decreases in aneuploidy rate should translate into decreases in spontaneous pregnancy loss. Two infertility centers utilizing DHEA supplementation, one in New York City and the other in Toronto, Canada, indeed, independently, reported identically low miscarriage rates of 15.0 and 15.2 percent, respectively. Depending on method of statistical analysis, these miscarriage rates represented declines of approximately 50 to 80 percent from expectations [10]. Even more remarkably, the combined loss rate of 15.1 percent equated rates reported for normal populations as young as 28 to 33 years [17], and was, thus, far removed from excessively high miscarriage rates, reported in DOR patients [18].

Even though such significant declines in spontaneous miscarriages cannot be achieved without underlying improvements in aneuploidy, miscarriage rates only represent surrogates for true aneuploidy studies. Direct evidence for such an effect was, therefore, still needed.

In this study we for the first time are able to demonstrate such direct evidence, utilizing routinely performed PGS of preimplantation stage embryos, performed in DHEA supplemented IVF cycles and controls. DHEA supplementation was, indeed, associated with significantly reduced aneuploidy, and greatest reductions were observed with short DHEA supplementation of up to 12 weeks.

This observation on first impulse suggests that, excluding month one of supplementation, second and third months offer the best chance of lowering aneuploidy, thus fully supporting previously published pregnancy data after DHEA supplementation, which demonstrated a significant first rise in pregnancy rate after approximately six weeks of DHEA supplementation [14]. Six weeks of DHEA supplementation prior to IVF cycle start, therefore, currently represents minimal supplementation time at our center.

This study, however, does not preclude, as alternative explanation for these findings that a more favorable patient group conceives quickly and, therefore, statistically distorts above noted time associations. Such a possibility cannot be ruled out since we previously demonstrated that women who improve AMH levels with DHEA supplementation demonstrate significantly superior pregnancy rates to those who do not [19].

A general criticism of currently available technologies for PGS is that only limited numbers of chromosomes can be evaluated (24 chromosome screening technologies are currently under investigation). In this study this meant that only seven chromosomes were assessed in study and control patients. This allows for the at least theoretical possibility that untested chromosomes demonstrate statistically different aneuploidy distributions from the here tested seven and that, including a full chromosome complement, here reported differences would disappear. Such an explanation is, however, highly unlikely, and the here utilized selection of chromosomes, or similar ones, have been routinely used in clinical PGS [15,16]. There is also no data in either human or animal literature to suggest that DOR maybe associated with aneuploidy of specific chromosomes.

Because of time pressures, when using their own oocytes, prospectively randomized clinical trials in patient populations affected by DOR, and involving placebo, are difficult, if not impossible, to conduct. Our center, for that reason, had to abandon two registered clinical DHEA trials, one in the United States and one in Europe, due to lack of enrollment [14]. A small first such trial has just been reported [3]. Best available evidence, therefore, at least in part, has to be obtained via other study formats.

In this study, the format chosen was a case control study in which each study patient/cycle was matched with two controls. As Table 1 demonstrates, patient and control populations appear, with few exceptions, overall comparable. It is, however, important to point out that the significantly larger preponderance of DOR in the study group (Table 1) biases study results against discovery of DHEA effects on ploidy since DOR patients demonstrate the highest aneuploidy rate amongst infertility patients [18]. Even just absence of increased aneuploidy in the study group could, therefore, be viewed as a potentially positive DHEA effect. Instead, this study actually demonstrates significantly lower aneuploidy following DHEA supplementation.

How DHEA affects non-dysfunctional events remains to be determined but we have speculated that DHEA supplementation may improve the ovarian environment in which follicular maturation takes place in older women [19 and submitted]. DHEA, indeed, significantly declines with advancing age [20]. Since DHEA, except in our prior pilot study [4], has never before been directly associated with decreases in aneuploidy, neither animal nor human data are currently available to speculate further on specific mechanisms that may be involved.

Others have speculated that drugs can be developed which beneficially affect non-dysjunctional events during meiosis [21]. DHEA may, indeed, turn out to be a first pharmacologic agent to do so.

This effect, only unlikely, should be restricted to infertile women with DOR. DHEA supplementation, in attempts to reduce embryo aneuploidy and spontaneous miscarriages, therefore, also deserves investigation in, especially older (above age 35 years) fertile women, attempting conception. A possible similar beneficial impact in fertile patient populations, attempting spontaneous conception, could have a major impact on public health by speeding up time to pregnancy and by reducing embryo aneuploidy and miscarriage rates.

## Abbreviations

AMH: anti-Müllerian hormone; DHEA: dehydroepiandrosterone; DOR: diminished ovarian reserve; FISH: fluorescence in situ hybridization; FSH: follicle stimulating hormone; GnRH-a: gonadotropins releasing hormone agonist; hMG: human menopausal gonadotropins; I.U.: international units; IVF: in vitro fertilization; mL: milliliter; T.I.D.: three-times daily;

## Competing interests

NG and DHB are listed as co-inventors on a number of U.S. patent applications (one already awarded), claiming therapeutic benefits from DHEA supplementation in women with diminished ovarian reserve (DOR). NG is owner of CHR, where this research was conducted. NG, DHB and AW received research grants, travel reimbursements and speaker honoraria from various pharmaceutical companies, none, however, related to DHEA or any other issues addressed in this manuscript. None of the authors has any formal links with pharmaceutical companies and/or owns shares in pharmaceutical companies.

## Authors' contributions

NG, AW and DHB contributed equally to the manuscript. NG, AW, DHB conceived and designed the study. AW conducted chart reviews. DHB performed statistical analyses. NG wrote manuscript. All three authors contributed, reviewed and approved the manuscript. All authors read and approved the final manuscript.

## References

[B1] Barad D Gleicher N Effect of dehydroepiandrosterone on oocytes and embryo yields, embryo grade and cell number in IVF Hum Reprod 2006 21 2845 2849 10.1093/humrep/del254 16997936

[B2] Barad D Brill H Gleicher N Update on the use of dehydroepiandrosterone supplementation among women with diminished ovarian function J Assist Reprod Genet 2007 24 629 634 10.1007/s10815-007-9178-x 18071895 PMC3454995

[B3] Wiser A Gonen O Ghetler Y Shavit T Berkovitz A Shulman A Addition of dehydroepiandrosterone (DHEA for poor-responder patients before and during IVF treatment improves the pregnancy rate: a randomized prospective study Hum Reprod 2010 25 2496 2500 10.1093/humrep/deq220 20729538

[B4] Gleicher N Weghofer A Barad D Increased euploid embryos after supplementation with dehydroepiandrosterone (DHEA) in women with premature ovarian aging Fertil Steril 2007 88 Suppl 1 S232 10.1016/j.fertnstert.2007.07.792

[B5] Eichenlaub-Ritter U Parentral age-related aneuploidy in human germ cells and offspring: a story of past and present Environ Mol Mutagen 1996 28 211 236 10.1002/(SICI)1098-2280(1996)28:3<211::AID-EM6>3.0.CO;2-G 8908181

[B6] Wyrobek AJ Aardema M Eichenlaub-Ritter U Ferguson L Marchetti F Mechanisms and targets involved in maternal and paternal age effects on numerical aneuploidy Environ Mol Mutagen 1996 28 254 264 10.1002/(SICI)1098-2280(1996)28:3<254::AID-EM9>3.0.CO;2-D 8908184

[B7] Twisk M Mastenbroek S van Wely M Heineman MJ Van der Veen F Repping S Preimplantation genetic screening for abnormal number of chromosomes (aneuploidies) in in vitro fertilization or intracytoplasmic sperm injection Cochrane Database Syst Rev 2006 25 1 CD005291 10.1002/14651858.CD005291.pub2 16437524

[B8] Gleicher N Weghofer A Barad D Preimplantation genetic screening: "established" and ready for prime time? Fertil Steril 2008 89 780 788 10.1016/j.fertnstert.2008.01.072 18353323

[B9] Mastenbroek S Twisk M van Echten-Arends J Sikkema-Raddatz B Korevaar JC Verhoeven HR Vogel NE Arts EG de Vries JW Bossuyt PM Buys CH Heineman MJ Repping S van der Veen F In vitro fertilization with preimplantation genetic screening N Engl J Med 2007 357 9 17 10.1056/NEJMoa067744 17611204

[B10] Gleicher N Ryan E Weghofer A Blanco-Mejia S Barad DH Miscarriage rates after dehydroepiandrosterone (DHEA) supplementation in women with diminished ovarian reserve: a case control study Reprod Biol Endocrinol 2009 7 108 10.1186/1477-7827-7-108 19811650 PMC2764711

[B11] Bettio D Venci A Levi Setti PE Chromosomal abnormalities in miscarriages after different assisted reproduction procedures Placenta 2008 29 Suppl B 126 8 10.1016/j.placenta.2008.08.015 18790324

[B12] Barad DH Weghofer A Gleicher N Age-specific levels for basal follicle-stimulatng hormone assessment of ovarian function Obstet Gynecol 2007 109 14014 1410 10.1097/01.AOG.0000264065.37661.a0 17540814

[B13] Barad DH Weghofer A Goyal A Gleicher N Age-specific anti-Müllerian hormone (AMH): Utility of AMH at various ages Reprod Biomed Online in press 10.1016/j.rbmo.2010.12.002 21269880

[B14] Barad D Brill H Gleicher N Update on the use of dehydroepiandrosterone supplementation among women with diminished ovarian function J Assist Reprod Genet 2007 24 629 634 10.1007/s10815-007-9178-x 18071895 PMC3454995

[B15] Weghofer A Munné S Brannath W Chen S Tomkin G Cekleniak N Garrisi M Barad D Cohen J Gleicher N The impact of LH-containing gonadotropins on diploidy rates in preimplantation embryos: long protocol stimulation Hum Reprod 2008 23 499 503 10.1093/humrep/dem412 18182396

[B16] Weghofer A Munné S Brannath W Chen S Barad D Cohen J Gleicher N The impact of LH-containing gonadotropins stimulation on euploidy rates in preimplantation embryos: antagonist cycles Fertil Steril 2009 92 937 942 10.1016/j.fertnstert.2008.07.1735 18774557

[B17] Herbert D Lucke J Dobson A Pregnancy losses in young Australian women: findings from the Australian Longitudinal Study of Women's Health Womens Health Issues 2009 19 21 29 10.1016/j.whi.2008.08.007 19111784

[B18] Levi AJ Raynault MF Bergh PA Drews MR Miller BT Scott RT Jr Reproductive outcome in patients with diminished ovarian reserve Fertil Steril 2001 76 666 669 10.1016/S0015-0282(01)02017-9 11591396

[B19] Gleicher N Weghofer A Barad DH Improvement in diminished ovarian reserve after dehydroepiandrosterone (DHEA) supplementation Reprod Biomed Online 2010 21 360 365 10.1016/j.rbmo.2010.04.006 20638339

[B20] Jacob MH da R Janner D Jahn MP Kucharski LC Belló-Klein A Ribeiro MF Age-related effects of DHEA on peripheral markers of oxidative stress Cell Biochem Funct 2010 28 52 57 10.1002/cbf.1619 19924683

[B21] Hodges CA Ilagan A Jenninger D Keri R Nilson J Hunt PA Experimental evidence that changes in oocyte growth influence meiotic chromosome segregation Hum Reprod 2002 17 1171 1180 10.1093/humrep/17.5.1171 11980735

